# Supplementary material for: Effects of Jumping Phase, Leading Limb, and Arena Surface Type on Forelimb Hoof Movement
Source: Animals (Basel). 2023 Jun 27;13(13):2122. doi: 10.3390/ani13132122 (PMC10340005; doi:10.3390/ani13132122)
Supplement: Supplementary file 1 [file animals-13-02122-s001.zip › animals-2406148-supplementary.pdf]

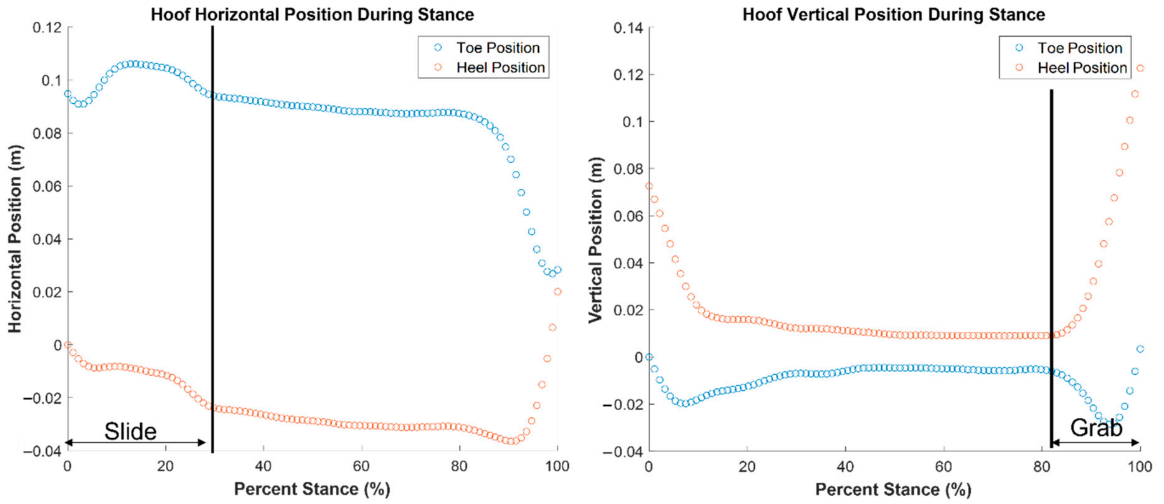

Figure S1. Subphases of stance: (**a**) Slide was defined as the region between the start of the stance phase and the end of horizontal hoof movement; (**b**) Grab was defined as the region near the end of stance where the toe sank deeper into the surface. Support captured the period of the stance phase between slide and grab, where horizontal and vertical hoof displacement were minimal. Graphs depict hoof movement for a single horse at landing.

Univariate Correlations between Surface Properties and Hoof Movement

Hoof displacement of the equine forelimb was linearly correlated with compositional variables (**Table S1**), manageable properties (**Table S2**), shear properties, and vertical impact properties (**Table S3**). Positive r-values between surface properties and hoof deceleration indicate greater hoof deceleration as the magnitude of that surface property increases. Positive r-values for horizontal translation and velocity are related to more movement in the direction of the center of mass of the horse, while positive r-values for vertical translation and velocity are related to more movement into the surface.

Table S1: Correlations (r-values) between compositional surface properties and hoof movement at takeoff and landing. Spearman correlations are noted with an asterisk (*); otherwise coefficients represent Pearson correlations.

**TAKEOFF**									
	Stance Phase	Fiber Content (%)	Sand Content (%)	Silt Content (%)	Clay Content (%)	Average Particle Size (mm)	Particle Size Deviation (mm)	Average Fiber Length (mm)	Fiber Length Deviation (mm)
Average Hoof Angle (°)	Slide	-*[a]	-*	-	-*	-*	-	-*	-*
	Support	-*	-*	-	-*	-*	-	-*	-*
	Grab	-*	-*	-	-0.32* (*P*=0.031) *n*=46	-*	-	-*	-*
Horizontal Toe Displacement (cm)	Slide	-*	-*	-	-*	-*	-	-*	-*
	Support	-*	-*	-*	-*	-*	-*	-*	-*
	Grab	-*	-*	-	-*	-*	-	-*	-*
Vertical Toe Displacement (cm)	Slide	-*	-*	-	-*	-*	-	-*	-*
	Support	-*	-*	-*	-*	-*	-*	-*	-*
	Grab	-*	-*	-	0.35* (*P*=0.016) *n*=46	0.33* (*P*=0.023) *n*=46	0.34 (*P*=0.020) *n*=46	-0.58* (*P*=0.002) *n*=27	-*
Horizontal Heel Displacement (cm)	Slide	-*	-*	-	-*	-*	-	-*	-*
	Support	-*	-*	-*	-*	-*	-0.29* (*P*=0.049) *n*=46	-*	-*
	Grab	-*	-*	-	-*	-0.31* (*P*=0.034) *n*=46	-0.30 (*P*=0.041) *n*=46	0.55* (*P*=0.003) *n*=27	-*
Vertical Heel Displacement (cm)	Slide	-*	-*	-	-0.40* (*P*=0.006) *n*=46	-0.31* (*P*=0.034) *n*=46	-	0.64* (*P*<0.001) *n*=27	-*
	Support	-*	-*	-	-*	-*	-	-*	-*
	Grab	-*	-*	-	0.42* (*P*=0.004) *n*=46	-*	-	-0.44* (*P*=0.021) *n*=27	-*

[a] -: Denotes that the correlation between variables was not statistically significant (*P*>0.05).

TAKEOFF									
	Stance Phase	Fiber Content (%)	Sand Content (%)	Silt Content (%)	Clay Content (%)	Average Particle Size (mm)	Particle Size Deviation (mm)	Average Fiber Length (mm)	Fiber Length Deviation (mm)
Average Horizontal Toe Velocity (m/s)	Slide	-*[a]	-*	-	-*	-*	-	-*	-*
	Support	-*	-*	-*	-*	-*	-*	-*	-*
	Grab	-*	-*	-*	-*	-*	-*	-*	-*
Average Vertical Toe Velocity (m/s)	Slide	0.33* (*P*=0.025) *n*=46	-*	-	-*	-*	-0.35 (*P*=0.017) *n*=46	-*	-*
	Support	-*	-*	-*	-*	-*	-*	-*	-*
	Grab	-*	-*	-	0.38* (*P*=0.009) *n*=46	0.33* (*P*=0.027) *n*=46	-	-0.56* (*P*=0.002) *n*=27	-*
Average Horizontal Heel Velocity (m/s)	Slide	-*	-*	-	-*	-*	-	NS*	-*
	Support	-*	-*	-*	-*	-*	-0.30* (*P*=0.041) *n*=46	NS*	-*
	Grab	-*	-*	-	-*	-*	-	0.52* (*P*=0.006) *n*=27	-*
Average Vertical Heel Velocity (m/s)	Slide	0.37* (*P*=0.012) *n*=46	-*	-	-0.38* (*P*=0.010) *n*=46	-0.42* (*P*=0.004) *n*=46	-0.46 (*P*=0.001) *n*=46	0.69* (*P*<0.001) *n*=27	-*
	Support	-*	-*	-	-*	-*	-	-*	-*
	Grab	-*	-*	-	-*	-*	-	-*	-*
Average Horizontal Toe Deceleration (m/s^2)	Slide	-*	0.31 (*P*=0.037) *n*=46	-	-*	-*	-	-*	-*
Average Horizontal Heel Deceleration (m/s^2)	Slide	-0.37* (*P*=0.012) *n*=46	-*	-	-*	0.33* (*P*=0.028) *n*=46	0.42 (*P*=0.004) *n*=46	-*	-*

[a] -: Denotes that the correlation between variables was not statistically significant (*P*>0.05).

LANDING									
	Stance Phase	Fiber Content (%)	Sand Content (%)	Silt Content (%)	Clay Content (%)	Average Particle Size (mm)	Particle Size Deviation (mm)	Average Fiber Length (mm)	Fiber Length Deviation (mm)
Average Hoof Angle (°)	Slide	-*[a]	-*	-	-*	-*	-	-*	-*
	Support	-*	-*	-	-*	-*	-	-*	-*
	Grab	-*	0.38*<br>(*P*=0.009)<br>*n*=46	-	-0.53*<br>(*P*<0.001)<br>*n*=46	-*	-	-*	-*
Horizontal Toe Displacement (cm)	Slide	-*	-*	-	-*	-*	-	-*	-*
	Support	-*	-*	-*	-*	-*	-*	-*	-*
	Grab	-*	-*	-	-*	-*	-	-*	-*
Vertical Toe Displacement (cm)	Slide	-*	-*	-	-0.42*<br>(*P*=0.004)<br>*n*=46	-*	-	-*	-*
	Support	-*	-*	-*	-*	-*	-*	-*	-*
	Grab	-*	-*	-	0.41*<br>(*P*=0.004)<br>*n*=46	0.30*<br>(*P*=0.042)<br>*n*=46	0.34<br>(*P*=0.023)<br>*n*=46	-*	-*
Horizontal Heel Displacement (cm)	Slide	-*	-*	-	-*	-*	-	-*	-*
	Support	-*	-*	-*	-*	-*	-*	-*	-*
	Grab	-*	-*	-	-0.36*<br>(*P*=0.015)<br>*n*=46	-0.30*<br>(*P*=0.043)<br>*n*=46	-0.34<br>(*P*=0.022)<br>*n*=46	0.46*<br>(*P*=0.017)<br>*n*=27	-*
Vertical Heel Displacement (cm)	Slide	-*	-*	-	-*	-*	-	-*	-*
	Support	-*	-*	-	-*	-*	-	-*	-*
	Grab	-*	-0.37*<br>(*P*=0.011)<br>*n*=46	-	0.54*<br>(*P*<0.001)<br>*n*=46	-*	-	-*	-*

[a] -: Denotes that the correlation between variables was not statistically significant (*P*>0.05).

LANDING									
	Stance Phase	Fiber Content (%)	Sand Content (%)	Silt Content (%)	Clay Content (%)	Average Particle Size (mm)	Particle Size Deviation (mm)	Average Fiber Length (mm)	Fiber Length Deviation (mm)
Average Horizontal Toe Velocity (m/s)	Slide	-*[a]	-*	-	-*	-*	-	-*	-*
	Support	-*	-*	-*	-*	-*	-*	-*	-*
	Grab	-*	-*	-*	-*	-*	-*	-*	-*
Average Vertical Toe Velocity (m/s)	Slide	-*	-*	-	-0.37* (P=0.011) n=46	-*	-	-*	-*
	Support	-*	-*	-*	-*	-*	-*	-*	-*
	Grab	-*	-*	-	0.42* (P=0.003) n=46	-*	0.33 (P=0.026) n=46	-*	-*
Average Horizontal Heel Velocity (m/s)	Slide	-*	-*	-	-*	-*	-	-*	-*
	Support	-*	-*	-*	-*	-*	-*	-*	-*
	Grab	-*	-*	-	-0.48* (P<0.001) n=46	-0.31* (P=0.039) n=46	-0.32 (P=0.029) n=46	0.45* (P=0.019) n=27	-*
Average Vertical Heel Velocity (m/s)	Slide	-*	-*	-	-*	-*	-0.29 (P=0.047) n=46	-*	-*
	Support	-*	-*	-	-*	-*	-	-*	-*
	Grab	-*	-0.38* (P=0.009) n=46	0.36 (P=0.014) n=46	0.43* (P=0.003) n=46	-*	-	-*	-*
Average Horizontal Toe Deceleration (m/s^2)	Slide	-*	-*	-	-*	-*	-	-*	-*
Average Horizontal Heel Deceleration (m/s^2)	Slide	-*	-*	-	-*	-*	-	-*	-*

[a] -: Denotes that the correlation between variables was not statistically significant (P>0.05).

Table S2: Correlations (r-values) between manageable surface properties and hoof movement at takeoff and landing. Spearman correlations are noted with an asterisk (*); otherwise coefficients represent Pearson correlations.

**TAKEOFF**				
	Stance Phase	Temperature (^{o}C)	Cushion Depth (mm)	Moisture Content (%)
Average Hoof Angle (^{o})	Slide	-*[a]	-	-*
	Support	-*	-	-*
	Grab	-*	-	-*
Horizontal Toe Displacement (cm)	Slide	-*	-	-*
	Support	-*	-*	-*
	Grab	-*	-	-*
Vertical Toe Displacement (cm)	Slide	-0.39* (*P*=0.007) *n*=46	-	-*
	Support	-*	0.33* (*P*=0.026) *n*=46	-*
	Grab	0.30* (*P*=0.040) *n*=46	-	-*
Horizontal Heel Displacement (cm)	Slide	-*	-	-*
	Support	-*	-*	-*
	Grab	-*	-	-*
Vertical Heel Displacement (cm)	Slide	-*	0.36 (*P*=0.015) *n*=46	-*
	Support	-*	-	-*
	Grab	-*	-	-*

[a] -: Denotes that the correlation between variables was not statistically significant (*P*>0.05).

TAKEOFF				
	Stance Phase	Temperature (°C)	Cushion Depth (mm)	Moisture Content (%)
Average Horizontal Toe Velocity (m/s)	Slide	-*[a]	-	-*
	Support	-*	-*	-*
	Grab	-*	-*	-*
Average Vertical Toe Velocity (m/s)	Slide	-0.48* (*P*<0.001) *n*=46	-	-*
	Support	-*	0.33* (*P*=0.025) *n*=46	-*
	Grab	-*	-	-*
Average Horizontal Heel Velocity (m/s)	Slide	-0.36* (*P*=0.013) *n*=46	-	-*
	Support	-*	-*	-*
	Grab	-*	-	-*
Average Vertical Heel Velocity (m/s)	Slide	-*	0.40 (*P*=0.006) *n*=46	-*
	Support	-*	-	-*
	Grab	-*	-	-*
Average Horizontal Toe Deceleration (m/s^2)	Slide	-*	-	-*
Average Horizontal Heel Deceleration (m/s^2)	Slide	-0.36 (*P*=0.014) *n*=46	-	-*

[a] -: Denotes that the correlation between variables was not statistically significant (*P*>0.05).

LANDING				
	Stance Phase	Temperature (°C)	Cushion Depth (mm)	Moisture Content (%)
Average Hoof Angle (°)	Slide	-*[a]	-	-*
	Support	-*	-	-*
	Grab	-*	-	-*
Horizontal Toe Displacement (cm)	Slide	-*	-	-*
	Support	-*	-	-*
	Grab	-*	-	-*
Vertical Toe Displacement (cm)	Slide	-*	-	-*
	Support	-*	-*	-*
	Grab	-*	-	-*
Horizontal Heel Displacement (cm)	Slide	-*	-	-*
	Support	-*	-*	-*
	Grab	-*	-	-0.29* (*P*=0.050) *n*=46
Vertical Heel Displacement (cm)	Slide	-*	-	-*
	Support	-*	-*	-*
	Grab	-*	-	-*

[a] -: Denotes that the correlation between variables was not statistically significant (*P*>0.05).

LANDING				
	Stance Phase	Temperature (°C)	Cushion Depth (mm)	Moisture Content (%)
Average Horizontal Toe Velocity (m/s)	Slide	-*[a]	-	-*
	Support	-*	-	-*
	Grab	-*	-	-*
Average Vertical Toe Velocity (m/s)	Slide	-*	-	-*
	Support	-*	-*	-*
	Grab	-*	-*	-*
Average Horizontal Heel Velocity (m/s)	Slide	-*	-	-*
	Support	-*	-*	-*
	Grab	-*	-	-*
Average Vertical Heel Velocity (m/s)	Slide	-*	-	-*
	Support	-*	-*	-*
	Grab	-*	-	-*
Average Horizontal Toe Deceleration (m/s^2)	Slide	-*	-	-*
Average Horizontal Heel Deceleration (m/s^2)	Slide	-*	-	-*

[a] -: Denotes that the correlation between variables was not statistically significant (*P*>0.05).

Shear Surface Properties and Hoof Movement: At takeoff horizontal toe deceleration was lower on surfaces with greater adhesion (r=-0.32; *P*=0.032), while at landing the heel had a greater vertical velocity on surfaces with greater adhesion (r=-0.41; *P*=0.006). No other hoof movement parameters were correlated with shear surface properties (adhesion, coefficient of friction, and maximum normalized shear force).

Table S3: Correlations (r-values) between vertical impact properties and hoof movement at takeoff and landing. Spearman correlations are noted with an asterisk (*); otherwise coefficients represent Pearson correlations.

**TAKEOFF**									
	Stance Phase	Maximum Vertical Impact Force (kN)	Impulse (N*s)	Loading Rate (kN/s)	Maximum Vertical Displacement (cm)	Soil Rebound (cm)	Dissipated Energy (J)	Stiffness (kN/m)	Maximum Deceleration (g)
Average Hoof Angle (°)	Slide	-[a]	-	-*	-	-	-	-*	-*
	Support	-	-	-*	-	-	-	-*	-*
	Grab	-	-	-*	-	-	-	-*	-*
Horizontal Toe Displacement (cm)	Slide	-	-	-*	-	-	-	-*	-*
	Support	-*	-*	-*	-*	-*	-*	-*	-*
	Grab	-	-	-*	-	-	-	-*	-*
Vertical Toe Displacement (cm)	Slide	-	-	-*	-	-	-	-*	-*
	Support	-*	-*	-*	-*	-*	-*	-*	-*
	Grab	-	-0.35 (*P*=0.018) *n*=45	-*	-	-	-	-*	-*
Horizontal Heel Displacement (cm)	Slide	-	-	-*	-	-	-	-*	-*
	Support	-*	-*	-*	-*	-*	-*	-*	-*
	Grab	-	-	-*	-	-	-	-*	-*
Vertical Heel Displacement (cm)	Slide	-	-	-*	-	0.38 (*P*=0.010) *n*=44	-	-*	-*
	Support	-	-	-*	-	-	-	-*	-*
	Grab	0.33 (*P*=0.026) *n*=45	-	-*	-	-	-	-*	-*

[a] -: Denotes that the correlation between variables was not statistically significant (*P*>0.05).

TAKEOFF									
	Stance Phase	Maximum Vertical Impact Force (kN)	Impulse (N*s)	Loading Rate (kN/s)	Maximum Vertical Displacement (cm)	Soil Rebound (cm)	Dissipated Energy (J)	Stiffness (kN/m)	Maximum Deceleration (g)
Average Horizontal Toe Velocity (m/s)	Slide	-[a]	-	-*	-	-	-	-*	-*
	Support	-*	-*	-*	-*	-*	-*	-*	-*
	Grab	-*	-*	-*	-*	-*	-*	-*	-*
Average Vertical Toe Velocity (m/s)	Slide	-	-	-*	-	-	-	-*	-*
	Support	-*	-*	-*	-*	-*	-*	-*	-*
	Grab	-	-	-*	-	-	-	-*	-*
Average Horizontal Heel Velocity (m/s)	Slide	-	-	-*	-	-	-	-*	-*
	Support	-*	-*	-*	-*	-*	-*	-*	-*
	Grab	-	-	-*	-	-	-	-*	-*
Average Vertical Heel Velocity (m/s)	Slide	-	0.33 (*P*=0.029) *n*=45	-*	-	0.46 (*P*=0.002) *n*=44	-	-*	-*
	Support	-	-	-*	-	-	-	-*	-*
	Grab	-	-	-*	-	-	-	-*	-*
Average Horizontal Toe Deceleration (m/s^2)	Slide	-	-	-*	-	-	-	-*	-*
Average Horizontal Heel Deceleration (m/s^2)	Slide	-	-	-*	-	-	-	-*	-*

[a] -: Denotes that the correlation between variables was not statistically significant ($P>0.05$).

LANDING									
	Stance Phase	Maximum Vertical Impact Force (kN)	Impulse (N*s)	Loading Rate (kN/s)	Maximum Vertical Displacement (cm)	Soil Rebound (cm)	Dissipated Energy (J)	Stiffness (kN/m)	Maximum Deceleration (g)
Average Hoof Angle (°)	Slide	-[a]	-	-*	-	-	-	-*	-*
	Support	-	-	-*	-	-	-	-*	-*
	Grab	-	-	-*	-	-	-	-*	-0.33* (*P*=0.026) *n*=45
Horizontal Toe Displacement (cm)	Slide	-	-	-*	-	-	-	-*	-*
	Support	-*	-*	-*	-*	-0.39* (*P*=0.010) *n*=44	-*	-*	-*
	Grab	-	-	-*	-	-	0.40 (*P*=0.007) *n*=44	-*	0.31* (*P*=0.039) *n*=45
Vertical Toe Displacement (cm)	Slide	-	0.31 (*P*=0.039) *n*=45	-*	-	-	-	-*	-*
	Support	-*	-*	-*	-*	-*	-*	-*	-*
	Grab	-	-0.30 (*P*=0.046) *n*=45	-*	-	-	-	-*	-*
Horizontal Heel Displacement (cm)	Slide	-	-	-*	-	-	-	-*	-*
	Support	-*	-*	-*	-*	-0.32* (*P*=0.034) *n*=44	-*	-*	-*
	Grab	-	-	-*	-	-	-	-*	-*
Vertical Heel Displacement (cm)	Slide	-	-	-*	-	-	-	-*	-*
	Support	-	-	-*	-	-	-	-*	-*
	Grab	0.38 (*P*=0.009) *n*=45	-	0.44* (*P*=0.023) *n*=45	-	-	0.30 (*P*=0.045) *n*=44	0.38* (*P*=0.011) *n*=44	0.40* (*P*=0.006) *n*=45

[a] -: Denotes that the correlation between variables was not statistically significant ($P>0.05$).

LANDING									
	Stance Phase	Maximum Vertical Impact Force (kN)	Impulse (N*s)	Loading Rate (kN/s)	Maximum Vertical Displacement (cm)	Soil Rebound (cm)	Dissipated Energy (J)	Stiffness (kN/m)	Maximum Deceleration (g)
Average Horizontal Toe Velocity (m/s)	Slide	-[a]	-	-*	-	-	-	-*	-*
	Support	-*	-*	-*	-*	-0.34* (*P*=0.024) *n*=44	-*	-*	-*
	Grab	-*	-*	-*	-*	-*	0.34* (*P*=0.026) *n*=44	-*	-*
Average Vertical Toe Velocity (m/s)	Slide	-	-	-*	-	-	-	-*	-*
	Support	-*	-*	-*	-*	-*	-*	-*	-*
	Grab	-	-	-*	-	-	-	0.31* (*P*=0.043) *n*=44	-*
Average Horizontal Heel Velocity (m/s)	Slide	-	-	-*	-	-	-	-*	-*
	Support	-*	-*	-*	-*	-*	0.31* (*P*=0.043) *n*=44	-*	-*
	Grab	-	-	-0.34* (*P*=0.023) *n*=45	-	-	-	-0.31* (*P*=0.043) *n*=44	-0.32* (*P*=0.035) *n*=45
Average Vertical Heel Velocity (m/s)	Slide	-	-	-*	-	-	-	-*	-*
	Support	-	-	-*	-	-	-	-*	-*
	Grab	-	-	-*	-	-	-	-*	0.38* (*P*=0.011) *n*=45
Average Horizontal Toe Deceleration (m/s^2)	Slide	-	-	-*	-	-	-	-*	-*
Average Horizontal Heel Deceleration (m/s^2)	Slide	-	-	-*	-	-	-	-*	-*

[a] -: Denotes that the correlation between variables was not statistically significant (*P*>0.05).

Table S4: Multivariate stepwise regression results between surface properties and hoof movement at takeoff and landing. Partial r values for each variable are reported, indicating the amount of variation in the dependent variable explained by each independent variable. For slide, positive values indicate more displacement or velocity in the direction of horse movement or into the surface. For support, positive values indicate more displacement or velocity opposite to the direction of horse movement or into the surface. For the toe during grab, positive values indicate more displacement or velocity opposite to the direction of horse movement or out of the surface. For the heel during grab, positive values indicate more displacement or velocity in the direction of horse movement or out of the surface.

**TAKEOFF**					
	Stance Phase	Independent Variable 1	Independent Variable 2	Independent Variable 3	Independent Variable 4
Average Hoof Angle (^{o})	Slide	-[a]			
	Support	Cushion Depth r=-0.35 (*P*=0.018)			
	Grab	-			
Horizontal Toe Displacement (cm)	Slide	-			
	Support	-			
	Grab	Maximum Deceleration r=-0.32 (*P*=0.041)			
Vertical Toe Displacement (cm)	Slide	-			
	Support	Temperature r=0.35 (*P*=0.023)	Cushion Depth r=0.44 (*P*=0.002)		
	Grab	Vertical Impact Impulse r=-0.40 (*P*=0.008)			
Horizontal Heel Displacement (cm)	Slide	-			
	Support	-			
	Grab	Particle Size Variation r=-0.32 (*P*=0.038)			
Vertical Heel Displacement (cm)	Slide	Average Fiber Length r=0.62 (*P*<0.001)	Soil Rebound r=0.39 (*P*=0.010)	Clay Content r=-0.33 (*P*=0.022)	
	Support	-			
	Grab	Maximum Deceleration r=-0.33 (*P*=0.027)	Average Fiber Length r=0.40 (*P*=0.041)		

[a] -: Denotes that the dependent variable was not related to any independent variables (*P*>0.05).

TAKEOFF					
	Stance Phase	Independent Variable 1	Independent Variable 2	Independent Variable 3	Independent Variable 4
Average Horizontal Toe Velocity (m/s)	Slide	-[a]			
	Support	-			
	Grab	-			
Average Vertical Toe Velocity (m/s)	Slide	Temperature r=-0.42 (*P*=0.005)			
	Support	Temperature r=0.41 (*P*=0.007)	Cushion Depth r=0.40 (*P*=0.004)		
	Grab	Particle Size Variation r=-0.33 (*P*=0.030)			
Average Horizontal Heel Velocity (m/s)	Slide	-			
	Support	-			
	Grab	-			
Average Vertical Heel Velocity (m/s)	Slide	Soil Rebound r=0.33 (*P*=0.012)	Clay Content r=-0.49 (*P*=0.004)		
	Support	-			
	Grab	-			
Average Horizontal Toe Deceleration (m/s^2)	Slide	Temperature r=-0.30 (*P*=0.047)			
Average Horizontal Heel Deceleration (m/s^2)	Slide	Particle Size Variation r=-0.41 (*P*=0.005)			

[a] -: Denotes that the dependent variable was not related to any independent variables (*P*>0.05).

LANDING					
	Stance Phase	Independent Variable 1	Independent Variable 2	Independent Variable 3	Independent Variable 4
Average Hoof Angle (°)	Slide	-[a]			
	Support	-			
	Grab	Clay Content r=-0.42 (*P*=0.004)			
Horizontal Toe Displacement (cm)	Slide	Adhesion r=0.33 (*P*=0.031)			
	Support	Dissipated Energy at Impact r=-0.32 (*P*=0.042)			
	Grab	Dissipated Energy at Impact r=-0.46 (*P*=0.002)			
Vertical Toe Displacement (cm)	Slide	Clay Content r=-0.33 (*P*=0.031)			
	Support	-			
	Grab	Particle Size Variation r=-0.37 (*P*=0.013)			
Horizontal Heel Displacement (cm)	Slide	Adhesion r=0.32 (*P*=0.041)			
	Support	Adhesion r=0.32 (*P*=0.030)			
	Grab	Clay Content r=-0.39 (*P*=0.009)			
Vertical Heel Displacement (cm)	Slide	-			
	Support	-			
	Grab	Clay Content r=-0.46 (*P*=0.002)	Vertical Loading Rate r=-0.28 (*P*=0.042)	Vertical Stiffness r=-0.33 (*P*=0.011)	Sand Content r=0.28 (*P*=0.019)

[a] -: Denotes that the dependent variable was not related to any independent variables ($P>0.05$).

LANDING					
	Stance Phase	Independent Variable 1	Independent Variable 2	Independent Variable 3	Independent Variable 4
Average Horizontal Toe Velocity (m/s)	Slide	Adhesion r=0.36 (*P*=0.015)			
	Support	Dissipated Energy at Impact r=-0.32 (*P*=0.041)			
	Grab	Dissipated Energy at Impact r=-0.37 (*P*=0.014)			
Average Vertical Toe Velocity (m/s)	Slide	Clay Content r=-0.45 (*P*=0.003)			
	Support	-[a]			
	Grab	Clay Content r=-0.42 (*P*=0.005)	Average Particle Size r=-0.28 (*P*=0.044)		
Average Horizontal Heel Velocity (m/s)	Slide	Adhesion r=0.36 (*P*=0.020)			
	Support	-			
	Grab	Clay Content r=-0.5 (*P*<0.001)			
Average Vertical Heel Velocity (m/s)	Slide	-			
	Support	-			
	Grab	Adhesion r=0.41 (*P*=0.005)			
Average Horizontal Toe Deceleration (m/s^2)	Slide	Adhesion r=0.33 (*P*=0.027)			
Average Horizontal Heel Deceleration (m/s^2)	Slide	Adhesion r=0.33 (*P*=0.027)			

[a] -: Denotes that the dependent variable was not related to any independent variables (*P*>0.05).
